# Randomized controlled trial of an Acceptance and Commitment Therapy and compassion-based group intervention for persons with inflammatory bowel disease: the LIFEwithIBD intervention

**DOI:** 10.3389/fpsyg.2024.1367913

**Published:** 2024-05-09

**Authors:** Cláudia Ferreira, Joana Pereira, David Skvarc, Sara Oliveira, Ana Galhardo, Nuno B. Ferreira, Paola Lucena-Santos, Sérgio A. Carvalho, Inês Matos-Pina, Bárbara S. Rocha, Francisco Portela, Inês A. Trindade

**Affiliations:** ^1^CINEICC, Faculty of Psychology and Education Sciences, University of Coimbra, Coimbra, Portugal; ^2^EMBRACE Lab, School of Behavioural, Social and Legal Sciences, University of Örebro, Örebro, Sweden; ^3^Instituto Superior Miguel Torga, Coimbra, Portugal; ^4^School of Social Sciences, University of Nicosia, Nicosia, Cyprus; ^5^HEI-Lab: Digital Human-Environment Interaction Lab, School of Psychology and Life Sciences, Lusófona University, Lisbon, Portugal; ^6^Center for Neuroscience and Cell Biology, Faculty of Pharmacy, University of Coimbra, Coimbra, Portugal; ^7^Coimbra University Hospital [CHUC], Gastroenterology Service, Coimbra, Portugal; ^8^School of Behavioural, Social and Legal Sciences, University of Örebro, Örebro, Sweden

**Keywords:** Acceptance and Commitment Therapy, compassion, inflammatory bowel disease, mindfulness, randomized controlled trial

## Abstract

**Objectives:**

This study tested the acceptability and efficacy of an Acceptance and Commitment Therapy and compassion-based intervention (LIFEwithIBD) in people with IBD through a two-arm RCT.

**Methods:**

Participants were recruited at the Gastroenterology Department of the Coimbra University Hospital between June and September 2019. Of the 355 patients screened, those who accepted to participate were randomly assigned to one of two conditions: experimental group (LIFEwithIBD; *n* = 25) or control group (waitlist; *n* = 29). Participants completed self-report measures at baseline (T0), post-intervention (T1), and 3-month (T2) and 12-month (T3) follow-ups. Intervention acceptability was assessed. Efficacy was examined using intent-to-treat ANCOVA at post-intervention after adjusting for baseline values of depressive, anxiety, and stress symptoms (primary outcomes). Linear mixed models for all longitudinal outcomes were also analysed. Inflammatory and disease biomarkers were determined at T0 and T3.

**Results:**

Acceptability results revealed a high level of satisfaction and perceived usefulness regarding the intervention. Both groups experienced a significant decrease in stress symptoms and IBD symptom perception at T1. No significant differences were observed at follow-up for the primary outcomes. The experimental group reported significantly lower Crohn’s disease Symptom severity at T2 than the control group. Post-hoc analyses designed to mitigate floor effects revealed substantial treatment effects for the experimental group regarding anxiety symptoms. No significant differences were observed in clinical biomarkers from T0 to T3.

**Conclusion:**

The LIFEwithIBD intervention shows promising, although preliminary, benefits for managing disease activity and reducing anxiety symptoms in IBD patients with high severity of psychological distress.

**Clinical trial registration**: https://www.clinicaltrials.gov/ct2/show/NCT03840707, identifier NCT03840707.

## 1 Introduction

Inflammatory bowel disease (IBD) considerably impacts the quality of life and raises the risk of developing mental health problems (Knowles et al., 2018). People with IBD have a higher prevalence of anxiety and depression than the general population (Barberio et al., 2021). Depression can increase IBD symptoms through the production of proinflammatory (Taché and Bernstein, 2009), which may itself increase levels of depressive symptoms (Keefer and Kane, 2017). In conjunction with medical care, psychological therapies might have beneficial effects on disease activity, mental health, and quality of life in people with IBD (Paulides et al., 2021).

Third-wave cognitive and behavioural therapies (CBT) (e.g., Mindfulness-Based Interventions, Acceptance and Commitment Therapy [ACT], and Compassion Focused Therapy [CFT]). have been suggested as particularly pertinent in populations with illness (Graham et al., 2016; Ferreira et al., 2018; Trindade et al., 2018). ACT intends to increase psychological flexibility through acceptance- and mindfulness-promoting techniques that increase willingness to go through adverse internal experiences (e.g., thoughts, feelings, sensations) while engaging with commitment in actions that promote a valued and meaningful life (Hayes et al., 2011).

Recent findings have highlighted that ACT-based interventions seem to be efficacious in reducing symptoms of stress (Lavelle et al., 2022), depression and anxiety (Romano et al., 2023) as well as feasible and well-accepted by adults living with IBD (Dober et al., 2021; Lavelle et al., 2022; Romano et al., 2023). Although these studies present limitations that limit the generalizability of results (e.g., small sample sizes) (Lavelle et al., 2022), they highlight the need for further research and replication (for example, through a full-scale randomized controlled trial) to further assess the efficacy of ACT-based interventions within the scope of IBD (Romano et al., 2023). To our knowledge, only two Randomized Controlled Trials (RCT) on ACT interventions have been performed in this population. These trials showed improvements in stress (Rowan et al., 2017; Wynne et al., 2019), depression, and general well-being (Wynne et al., 2019) compared to a control group.

Mindfulness-based interventions that aim to promote present-moment awareness and a non-judgmental attitude towards internal experiences have been tested in the context of IBD (Ewais et al., 2019). Mindfulness-Based Stress Reduction (MBSR) interventions have been shown to increase the quality of life (Jedel et al., 2014; Goren et al., 2022) and reduce psychological symptoms (Drent et al., 2016; Goren et al., 2022), fatigue (Goren et al., 2022), and the concentration of C-reactive Protein (CRP) and faecal calprotectin in IBD (Berrill et al., 2014; Gonzalez-Moret et al., 2020).

Compassion-focused therapy addresses aspects such as shame, stigma and self-criticism, which are often reported by people with IBD and influence their psychosocial functioning (Taft et al., 2017; Trindade et al., 2020a). Self-compassion, defined as the sensitivity and the desire to alleviate one’s suffering and the ability to extend kindness and understanding towards the self when facing personal setbacks or inadequacies, has been shown to have a protective effect against psychological distress in IBD (Trindade and Sirois, 2021). The integration of compassion-based components in psychological interventions in this population may thus be beneficial and has been proven effective in other chronic conditions (Brown et al., 2019; Gooding et al., 2020).

Previous studies that tested the efficacy of integrative programs based on self-compassion components, ACT and mindfulness-based in chronic health conditions appear to be feasible and efficacious in improving quality of life, mental health, psychological flexibility, or shame (Skinta et al., 2015; Trindade et al., 2020b). Nonetheless, this kind of integrative interventions have yet to be tested in IBD.

The present RCT tests the acceptability and preliminary efficacy of a face-to-face intervention tailored for IBD patients, the “Living with Intention, Fullness, and Engagement with Inflammatory Bowel Disease” intervention (Trindade et al., 2021), in comparison with a waitlist control condition (“psychological treatment as usual” in Portugal). This study’s main research questions relate to whether the LIFEwithIBD intervention presents acceptability among participants and efficacy in improving psychological distress when compared to the control condition. It is expected that the LIFEwithIBD intervention will be well-accepted by participants and will lead to decreases in anxiety and depression, over the ones that might be reported by the control group.

## 2 Methods and materials

### 2.1 Study design

A detailed study protocol with a session-by-session description of the LIFEwithIBD intervention, its implementation, and methodology for the current RCT is available in open access elsewhere (Trindade et al., 2020b). The planning and implementation of this study respected the ethical recommendations by the American Psychological Association and the World Medical Association’s Declaration of Helsinki. Ethical approval was obtained from the Portuguese Data Protection Authority and the Research Ethics Committee of the Coimbra Hospital and University Center (CHUC).

### 2.2 Participants

#### 2.2.1 Participants’ recruitment and selection

**Figure 1:**
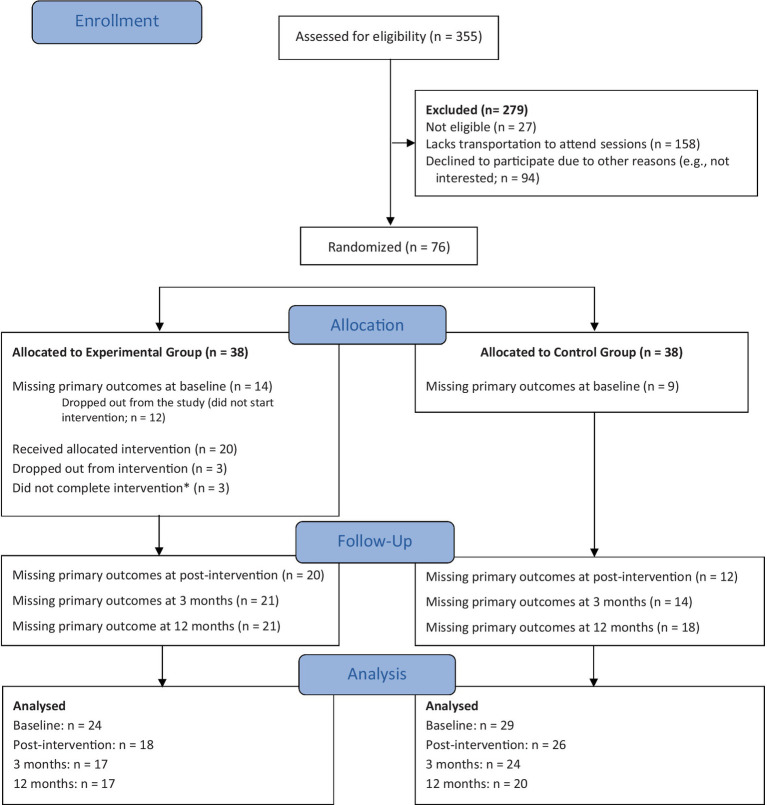
CONSORT flow diagram. ^*^Participants with three consecutive absences or who attended less than two-thirds of the intervention were considered intervention non-completers.

Participants were recruited from June to September 2019 at the Gastroenterology Department of the Centro Hospitalar e Universitário de Coimbra (Coimbra University Hospital). A total of 355 patients were screened by psychologists from the research team through an in-person eligibility interview (Figure 1). Inclusion criteria were: (a) 18 to 65 years old, (b) being able to read and write Portuguese, (c) having an IBD diagnosis for at least 6 months, and (d) being able to give informed consent. Exclusion criteria were: (a) having started a new treatment for IBD in the previous 6 months (in the case of anti-TNF and immunosuppressive therapy) or 2 months (in the case of steroid or aminosalicylate therapy), (b) presenting a diagnosed psychiatric disorder (major depressive disorder, psychotic disorder, bipolar disorder, substance abuse), severe depression, or suicidal ideation (assessed by the Patient Health Questionnaire-9)1, (c) undergoing any other form of psychological intervention, and (d) current pregnancy. Patients who were non-eligible due to exclusion criteria (b) were referred to national mental healthcare services. Please see the RCT’s study protocol for a more detailed presentation of the study’s recruitment and allocation (Trindade et al., 2021).

#### 2.2.2 Participants’ description

A total of 76 participants were selected and randomly assigned to one of two conditions: the experimental group (EG; LIFEwithIBD + TAU: *n* = 38) or the control group (CG; TAU: *n* = 38) (Figure 1). Before the intervention began for the EG, all participants were contacted by phone. In this process, 23 participants (14 for the EG and 9 for the CG) self-excluded themselves from the study due to a lack of resources to attend the sessions (e.g., lack of time or transportation) or loss of contact.

The sociodemographic and clinical characteristics of the sample are presented in Table 1.

**Table 1:** Demographic and clinical characteristics of the study groups at baseline.

		Experimental group (*n* = 24)	Control group (*n* = 29)
Time since diagnosis (in years), M (SD)		9.96 (7.64)	12.50 (8.18)
IBD diagnosis, *n* (%)	Crohn’s Disease	14 (58.30)	15 (51.72)
	Ulcerative Colitis	10 (41.70)	14 (48.28)
Gender, *n* (%)	Man	11 (45.80)	13 (44.80)
	Woman	13 (54.20)	16 (55.2)
Marital status, *n* (%)	Single	12 (50.00)	6 (20.69)
	Married/living with a partner	10 (41.70)	21 (72.41)
	Divorced	0 (0)	2 (6.90)
	widower	2 (8.30)	0 (0)
Place of residence, *n* (%)	Urban	20 (83.30)	23.00 (79.31)
	Rural	4 (16.70)	6 (20.69)
Education, *n* (%)	1–4 years	0 (0)	0 (0)
	5–6 years	0 (0)	3 (10.34)
	7–9 years	2 (8.30)	0 (0)
	10–12 years	13 (54.20)	13 (44.83)
	Bachelor’s	5 (20.8)	9 (31.03)
	Master’s	3 (12.50)	2 (6.90)
	PhD	0 (0)	2 (6.90)
	Other post-graduate degree	1 (4.2)	0 (0)
DASS depression, *n* (%)	Normal	17 (68.00)	20 (69.00)
	Mild	2 (8.00)	3 (10.30)
	Moderate	2 (8.00)	3 (10.30)
	Severe	2 (8.00)	2 (6.90)
	Extremely severe	2 (8.00)	1 (3.40)
DASS anxiety, *n* (%)	Normal	17 (68.00)	24 (82.80)
	Mild	3 (12.00)	1 (3.40)
	Moderate	4 (16.00)	3 (10.30)
	Severe	1 (4.00)	0 (0)
	Extremely severe	0 (0)	1 (3.40)
DASS stress, *n* (%)	Normal	7 (29.20)	16 (55.20)
	Mild	3 (12.50)	1 (3.40)
	Moderate	7 (29.20)	6 (20.70)
	Severe	4 (16.70)	3 (10.30)
	Extremely severe	3 (12.50)	3 (10.30)

#### 2.2.3 Measures

Participants completed the following self-reported measures at T0 (baseline), T1 (post-treatment), T2 (3 months follow-up), and T3 (1-year follow-up):

- Primary outcome:
- Psychological distress (DASS-21)
- Secondary outcomes:
  - Functional impairment (WSAS).
  - General quality of life (EUROHIS-QOL 8-item index).
  - Health-related quality of life (IBDQ-UK).
  - Chronic illness-related shame (CISS).
  - IBD symptom perception (IBD symptoms scale).
  - Disease activity: Mayo Score, Harvey-Bradshaw Score.
  - Self-Compassion (SCS).
  - Psychological flexibility (CompACT).
  - Biomarkers complete blood count with erythrocytic indexes, serum albumin, C Reactive Protein (CRP) and faecal calprotectin. Differences of each biomarker were analysed at T0 and T3.
- To assess the intervention’s acceptability, participants in the EG answered a questionnaire post-intervention regarding the following topics:
- Quality of intervention (1 = *poor* to 10 = *excellent*);
- Usefulness of intervention (1 = *nothing useful* to 10 = *extremely useful*);
- Individual perception of change during and after the intervention - physical symptoms related to IBD, emotion regulation skills, and quality of life (1 = *no difference* to 10 = *much better*);
- How do significant others (e.g., family, friends) perceive changes in terms of calmness, kindness, happiness, and emotional stability (1 = *much less* to 10 = *much more*);
- Recommendation of the intervention to other people with IBD (*yes/no*);
- Personal comment or experience about intervention (open response).

Each instrument is described in detail in this RCT’s study protocol (Trindade et al., 2021), which also presents the study’s Recommendations for Interventional Trials (CONSORT) figure.

#### 2.2.4 Data analysis

Descriptive statistics (means, standard deviations, and frequencies) were computed for characterisation purposes and to assess the acceptability of the intervention. The primary outcome was psychological distress (DASS-21 - depression, anxiety, and stress) measured at 9 weeks (immediately after intervention) between the two intervention groups and adjusting for baseline scores using ANCOVA. We examined the ANCOVA’s assumptions through standard tests and residual plots, and no transformations or corrections were required. We then calculated the magnitude of the differences between treatment groups as the standardised mean difference. Between-groups’ differences at post-treatment were examined for the following continuous outcomes: IBD symptom perception (IBDSS); psychological processes (SCS, CompACT); chronic illness-related shame (CISS); work and social adjustment (WSAS); quality of life (EUROHIS-QOL-8, IBDQ-UK), and disease activity (Mayo Score for ulcerative colitis participants; and Harvey-Bradshaw Score for Crohn’s Disease participants). All analyses were conducted using ANCOVA with baseline values as a covariate, and all analyses were intent-to-treat. All analyses were performed using the *car packages* (Fox et al., 2012) and *emmeans* (Lenth et al., 2018) in R and GAMLj in Jamovi.

##### 2.2.4.1 Longitudinal outcomes

We used Mixed Models of Repeated Measures (MMRM) to examine for Condition*Time interaction effects on each of the continuous outcomes from baseline, 9 weeks, 3 and 12 months. The Satterthwaite method for degrees of freedom was used for all linear models. We attempted to fit polynomial contrast estimates for the repeated linear, quadratic, and cubic measurements.

##### 2.2.4.2 Exploratory post-hoc analyses

Given the exploratory nature of the pilot study, we persisted with our interrogation of the data even in the absence of statistically significant omnibus main effects or interactions. No correction was made for post-hoc multiple comparisons. To account for the possibility of floor effects due to generally low levels of psychological distress symptoms, we performed additional exploratory analyses of the primary outcomes, excluding participants who did not at least report mild symptoms at T0.

The clinical significance of changes of each participant was analysed by computing the reliable change index (RCI) and considering mixed models of repeated measures.

##### 2.2.4.3 Biomarkers

The results are expressed as the mean ± SEM of the values in each group. Two-way ANOVA followed by Sidak’s multiple comparison test was used to compare faecal calprotectin, CRP, albumin and haematological parameters between T0 and T3. A probability value (*p*) of less than 0.05 was considered significant. Statistical analysis was performed using GraphPad prism software version 9.0.

##### 2.2.4.4 Data integrity

Please see Supplementary material.

## 3 Results

### 3.1 Acceptability assessment

An overview of the acceptability of the LIFEwithIBD intervention is presented in Table 2. In general, most participants from the EG considered the intervention to be of high quality and useful. Participants reported perceived changes in IBD-related physical symptoms, psychological well-being outcomes, and quality of life after the intervention. Also, participants indicated that significant others (e.g., spouse, family, friends) identified positive changes regarding calmness, kindness, and happiness. All participants stated that the LIFEwithIBD intervention had been worthwhile and that they would recommend it to another person with IBD. Some participants left statements about the intervention (Table 3).

**Table 2:** Acceptability evaluation of the LIFEwithIBD intervention (experimental group; *n* = 18).

	M (SD)	Range
*LIFEwithIBD intervention*		
Quality of the intervention	9.17 (1.51)	5–10
Usefulness of the intervention	9.17 (1.58)	5–10
*Participants’ perceived change*		
Degree of change in physical symptoms associated with IBD	6.11 (2.47)	1–10
Degree of change in emotion regulation skills and quality of life	7.50 (1.98)	4–10
Extent to which family/friends/co-workers notice participant is more calm/relaxed	7.06 (1.92)	2–10
Extent to which family/friends/co-workers notice participant is kinder	6.56 (1.79)	2–10
Extent to which family/friends/co-workers notice participant is happier	6.67 (1.85)	2–10
Extent to which family/friends/co-workers notice participant is more emotionally stable	6.94 (1.86)	2–10

	Yes (*n*, %)	No (*n*, %)
Would recommend the LIFEwithIBD intervention to another person with IBD	18, 100	0, 0

**Table 3:** Patient statements about the LIFEwithIBD intervention.

*“It was very important for me to participate in this study because my life was not at a good moment, and all the skills I gained in this study gave me the strength to change some aspects of myself. Furthermore, I know that all the help from the experts was the best, and I really appreciate all the time they gave us.”* (21-year-old participant)
*“I am very sorry that [the intervention] has ended. I’ve continued to use the exercises, even more now because of the isolation due to the Covid-19 pandemic. I feel strengthened.”* (44-year-old participant)
*“I value the present moment more now. My stuttering decreased, and I feel better about myself.”* (48-year-old participant)
*“It was a good initiative, and I hope that they [the researchers/therapists] can continue with other groups and that this will be a great help for developments in IBD.”* (30-year-old participant)
*“Congratulations on the intervention. I hope it is extended to all who suffer from these diseases. We will all certainly improve.”* (48-year-old participant)

### 3.2 Efficacy assessment

No substantial differences at T0 for any demographic variable were observed, but significant differences between groups for anxiety, self-critical attitude, and psychological flexibility were found (Table 4).

**Table 4:** Analysis of simple effects.

Outcome	Time	Group	Time	Group	*MD*	*SE*	*t*	*DF*	*p*-value	Effect size CI 95%
Anxiety^a^	1	Experimental	3	Experimental	−2.12	0.74	−2.872	119	0.005**	SMD = −0.91 [−1.5, −0.26]
	0	Experimental	1	Experimental	1.77	0.71	2.496	131	0.014*	SMD = 0.78 [0.14, 1.4]
	0	Experimental	0	Control	1.73	0.84	2.057	104	0.042*	SMD = 0.67 [0.01, 1.3]
	3	Experimental	3	Control	2.75	0.96	2.851	131	0.005**	SMD = 0.95 [0.25, 1.6]
Stress^a^	0	Experimental	1	Experimental	2.26	0.93	2.41	128.7	0.017*	SMD = 0.76 [0.12, 1.4]
	3	Experimental	3	Control	3.05	1.40	2.17	113.2	0.031*	SMD = 0.75 [0.05, 1.4]
IBD Symptom perception^b^	0	Control	1	Control	4.92	2.39	2.05	118.5	0.042*	SMD = 0.64 [0.01, 1.2]
	0	Experimental	1	Experimental	7.08	2.9	2.44	126.5	0.016*	SMD = 0.83 [0.13, 1.5]
	0	Experimental	3	Experimental	7.15	2.92	2.45	124.3	0.016*	SMD = 0.84 [0.13, 0.15]
Self-compassionate attitude^c^	0	Experimental	2	Experimental	−0.27	0.13	−2.029	124.1	0.045*	SMD = 0.69 [0.002, 1.38]
Self-critical attitude^c^	0	Experimental	0	Control	0.47	0.20	2.339	70.3	0.022*	SMD = 0.79 [0.10, 1.4]
	1	Experimental	1	Control	0.44	0.21	2.11	80.9	0.038*	SMD = 0.69 [0.03, 1.36]
	2	Experimental	2	Control	0.58	0.21	2.738	84.3	0.008**	SMD = 0.93 [0.22, 1.64]
	3	Experimental	3	Control	0.73	0.21	3.357	88.3	0.001**	SMD = 1.15 [0.42, 1.87]
Psychological flexibility^d^	0	Experimental	0	Control	−11.89	4.07	−2.924	74.6	0.005*	SMD = 0.92 [0.29, 1.57]
	1	Experimental	1	Control	−9.43	4.29	−2.196	86.7	0.031*	SMD = 0.65 [0.05, 1.33]
	2	Experimental	2	Control	−12.91	4.36	−2.963	90.7	0.004**	SMD = 0.93 [0.28, 1.58]
	3	Experimental	3	Control	−12.03	4.43	−2.715	95.3	0.008*	SMD = 0.85 [0.20, 1.5]

^a^Depression Anxiety Stress Scales (DASS-21); ^b^IBD symptoms scale (IBDSS); ^c^Self-Compassion Scale (SCS); ^d^Comprehensive Assessment of Acceptance and Commitment Therapy Processes (CompACT). T0, BASELINE; T1, POST-TREATMENT; T2, 3-MONTH FOLLOW-UP; T3, 6-MONTH FOLLOW-UP.

*p < 0.05, **p < 0.01.

#### 3.2.1 Primary outcome analyses

We observed no significant differences between groups at T1 on any of the DASS sub-scales after adjusting for T0 symptom scores.

#### 3.2.2 Secondary outcome analyses

In every outcome examined, T0 symptom scores significantly predicted symptom scores at T1 (or T2 for the Harvey-Bradshaw or Mayo score). In contrast, group membership in either experimental or control conditions was almost entirely unrelated to follow-up scores, with the sole exception of the Harvey-Bradshaw score. Once the T2 Harvey-Bradshaw severity was adjusted for baseline values, the EG reported significantly fewer symptoms than the CG (*η_p_^2^* = 0.155, *p* = 0.042; Table 5).

**Table 5:** Primary and secondary outcome analysis.

	Experimental group				Control group				ANCOVA
	T0		T1		T0		T1		
	M	SD	M	SD	M	SD	M	SD	
Depression^a^	4.54	4.97	3.11	2.68	3.20	4.05	3.19	4.33	T0: *F* (1, 40) = 20.35, *p* < 0.001.
									Group: *F* (1, 40) = 1.19, *p* = 0.282
Anxiety^a^	4	3.31	2.41	2.20	2.37	3.509	2.46	2.66	T0: *F* (1, 40) = 16.617, *p* < 0.001.
									Group: *F* (1, 40) = 1.496, *p* = 0.229
Stress^a^	7.87	4.71	5.82	3.24	5.72	4.94	4.88	4.44	T0: *F* (1, 40) = 19.519, *p* < 0.001.
									Group: *F* (1, 40) = 0.16, *p* = 0.901
Harvey Bradshaw score*	1.36	1.27	0.93	1.27	1.57	1.5	1.71	1.54	T0: *F* (1, 25) = 64.67, *p* < 0.001.
									Group: *F* (1, 25) = 4.59, *p* = 0.042.
Mayo score – stool frequency	1	1.24	0.80	1.13	0.71	1.36	0.71	0.989	T0: *F* (1, 21) = 84.01, *p* < 0.001.
									Group: *F* (1, 21) = 0.37, *p* = 0.546
Mayo score – rectal bleeding	0.40	0.843	0.40	0.843	0.14	0.343	0.14	0.363	T0: *F* (1, 21) = 69.8, *p* < 0.001.
									Group: *F* (1, 21) = 0.063, *p* = 0.805
Mayo score – physician rating	0.70	0.825	0.70	0.823	0.50	0.855	0.50	0.76	T0: *F* (1, 21) = 142.8, *p* < 0.001
									Group: *F* (1, 21) = 0.046, *p* = 0.883
IBD symptom perception^b^	37.37	14.95	32.00	12.68	30.48	19.25	26.62	14.18	T0: *F* (1, 40) =51.957, *p* < 0.001
									Group: *F* (1, 40) =0.062, *p* = 0.805
Self-compassionate attitude^c^	3.09	0.73	3.14	0.76	3.19	0.76	3.28	0.64	T0: *F* (1, 40) =19.499, *p* < 0.001
									Group: *F* (1, 40) =0.012, *p* = 0.913
Self-critical attitude^c^	2.71	0.73	2.8	0.81	2.29	0.68	2.3	0.66	T0: *F* (1, 40) =43.71, *p* < 0.001
									Group: *F* (1, 40) =0.311, *p* = 0.58
Psychological flexibility^d^	57.92	10.97	58.33	14.69	69.66	13.63	67.77	17.23	T0: *F* (1, 40) =40.881, *p* < 0.001
									Group: *F* (1, 40) =0.178, *p* = 0.675
Chronic illness-related shame^e^	9.33	6.16	9.83	5.14	6.66	5.68	6.15	5.36	T0: *F* (1, 40) =63.711, *p* < 0.001
									Group: *F* (1, 40) =0.886, *p* = 0.352
Functional impairment^f^	12.54	8.4	12.94	8.08	8.34	8.77	10.42	10.59	T0: *F* (1, 40) =45.717, *p* < 0.001
									Group: *F* (1, 40) =0.041, *p* = 0.841
General quality of life ^g^	62.92	12.83	60.94	10.01	65.41	16.66	65.63	17.7	T0: *F* (1, 40) =92.255, *p* < 0.001
									Group: *F* (1, 40) =0.013, *p* = 0.911
Health-related quality of life^h^	28.94	11.38	29.27	12.18	26.44	13.39	25.68	12.51	T0: *F* (1, 40) =79.871, *p* < 0.001
									Group: *F* (1, 40) =0.799, *p* = 0.377

*Harvey Bradshaw and Mayo Scores are for the baseline (T0) and the post-treatment follow up (T2).

^a^Depression Anxiety Stress Scales (DASS-21); ^b^IBD symptoms Scale (IBDSS); ^c^Self-Compassion Scale (SCS); ^d^Comprehensive Assessment of Acceptance and Commitment Therapy Processes (CompACT); ^e^Chronic illness-related shame Scale (CISS); ^f^Work and Social Adjustment Scale (WSAS); ^g^EUROHIS-QOL-8; ^h^Inflammatory Bowel Disease Questionnaire (IBDQ-UK).

We observed negative linear effects of time for stress symptoms and IBD symptom perception that did not differ between the groups and a weak positive quadratic effect of time for self-compassionate attitude. We observed between groups effects for self-critical attitude and psychological flexibility, with the EG reporting a poorer function in both (Table 6).

**Table 6:** Longitudinal analyses—omnibus effects for all outcomes.

	R^2^	Time				Group				Time * Group			
		*F*	*dfT*	*dfR*	*p*-value	*F*	*dfT*	*dfR*	*p*-value	*F*	*dfT*	*dfR*	*p*-value
Depression^a^	0.03	1.237	3	119.5	0.300	1.659	1	49.3	0.204	0.844	3	119.5	0.472
Anxiety^a^	0.04	1.44	3	122.5	0.233	4.28	1	51.9	0.044*	2.68	3	122.5	0.050
Stress^a^	0.07	2.853	3	120.2	0.040*	3.466	1	51.8	0.068	0.817	3	120.2	0.487
IBD symptom perception^b^	0.05	4.153	3	119.3	0.008**	1.64	1	52.2	0.206	0.26	3	119.3	0.854
Self-compassionate attitude^c^	0.01	1.859	3	115.4	0.140	0.138	1	48.5	0.712	0.32	3	115.4	0.811
Self-critical attitude^c^	0.12	0.0483	3	118.5	0.986	8.8554	1	52.7	0.004**	1.2763	3	118.5	0.286
Psychological flexibility^d^	0.14	2.123	3	120.5	0.101	9.635	1	54.1	0.003**	0.38	3	120.5	0.767
Chronic illness-related shame^e^	0.06	1.712	3	117.7	0.168	3.633	1	51	0.062	0.322	3	117.7	0.809
Functional impairment^f^	0.05	0.904	3	120.2	0.441	3.255	1	52.5	0.077	0.116	3	120.2	0.950
General quality of life^g^	0.02	1.202	3	116.4	0.312	1.294	1	50.7	0.261	0.318	3	116.4	0.812
Health-related quality of life^h^	0.01	0.841	3	120.3	0.474	0.39	1	53.5	0.535	0.621	3	120.3	0.603

^a^Depression Anxiety Stress Scales (DASS-21); ^b^IBD symptoms scale (IBDSS); ^c^Self-Compassion Scale (SCS); ^d^Comprehensive Assessment of Acceptance and Commitment Therapy Processes (CompACT); ^e^Chronic illness-related shame Scale (CISS); ^f^Work and Social Adjustment Scale (WSAS); ^g^EUROHIS-QOL-8; ^h^Inflammatory Bowel Disease Questionnaire (IBDQ-UK).

* *p* < 0.05, ** *p* < 0.01.

Finally, we observed a single time by group quadratic interaction for anxiety symptoms, where the EG demonstrated an improvement from T0 to T1 but then stabilised at baseline levels (Table 7).

**Table 7:** Longitudinal outcomes—fixed effects parameters.

	Depression^a^				Anxiety^a^				Stress^a^			
	*B*	*df*	*t*	*p*-value	*B*	*df*	*t*	*p*-value	*B*	*df*	*t*	*p*-value
(Intercept)	3.344	49.3	6.938	< 0.001	2.902	51.9	8.279	< 0.001	6.198	51.8	11.269	< 0.001
Time (Linear)	−0.954	124.3	−1.562	0.121	0.028	126.1	0.083	0.934	−1.696	124.5	−2.795	0.006**
Time (Quadratic)	−0.522	123.2	−0.839	0.403	0.534	122.1	1.567	0.120	−0.351	123.3	−0.570	0.570
Group	−1.242	49.3	−1.288	0.204	−0.725	51.9	−2.069	0.044*	−2.048	51.8	−1.862	0.068
Time*Group (Linear)	1.530	124.3	1.252	0.213	−0.484	126.1	−1.411	0.161	1.136	124.5	0.936	0.351
Time*Group (Quadratic)	0.007	123.2	0.006	0.995	−0.796	122.1	−2.337	0.021*	0.738	123.3	0.598	0.551

	IBD symptom perception^b^				Self-compassionate attitude^c^				Self-critical attitude^c^			
	*B*	*df*	*t*	*p*-value	*B*	*df*	*t*	*p*-value	*B*	*df*	*t*	*p*-value
(Intercept)	30.100	52.2	15.449	< 0.001	3.237	48.5	35.279	< 0.001	2.518	52.7	26.747	< 0.001
Time (Linear)	−6.000	123.0	−3.198	0.002**	0.162	119.3	1.863	0.065	−0.028	121.5	−0.365	0.716
Time (Quadratic)	−3.870	121.9	−2.029	0.045*	0.191	118.1	2.165	0.032*	−0.008	120.6	−0.097	0.923
Group	−4.990	52.2	−1.281	0.206	0.068	48.5	0.371	0.712	−0.560	52.7	−2.976	0.004**
Time*(Linear)	2.160	123.0	0.576	0.566	−0.038	119.3	−0.217	0.828	0.026	121.5	0.170	0.866
Time *(Quadratic)	2.220	121.9	0.582	0.562	−0.166	118.1	−0.942	0.348	−0.115	120.6	−0.737	0.462

	Psychological flexibility^d^				Chronic illness-related shame^e^				Functional impairment^f^			
	*B*	*df*	*t*	*p*-value	*B*	*df*	*t*	*p*-value	*B*	*df*	*t*	*p*-value
(Intercept)	65.397	54.1	35.093	< 0.001	7.777	51.0	11.128	< 0.001	10.423	52.5	9.476	< 0.001
Time (Linear)	0.302	123.9	0.181	0.857	−0.634	121.2	−0.998	0.320	0.981	124.2	0.865	0.388
Time (Quadratic)	2.952	122.8	1.737	0.085	−1.013	120.1	−1.569	0.119	−0.712	123.0	−0.618	0.538
Group	11.569	54.1	3.104	0.003**	−2.664	51.0	−1.906	0.062	−3.969	52.5	−1.804	0.077
Time*Group (Linear)	−2.469	123.9	−0.738	0.462	−0.344	121.2	−0.270	0.787	1.182	124.2	0.521	0.603
Time*Group (Quadratic)	1.014	122.8	0.299	0.766	0.890	120.1	0.690	0.492	0.388	123.0	0.168	0.867

	Health-related quality of life^g^				General quality of life^h^			
	*B*	*df*	*t*	*p*-value	*B*	*df*	*t*	*p*-value
(Intercept)	26.481	53.5	16.791	< 0.001	65.572	50.7	34.459	< 0.001
Time (Linear)	−0.863	123.9	−0.580	0.563	1.972	119.4	1.305	0.194
Time (Quadratic)	−2.381	122.8	−1.575	0.118	2.758	118.4	1.798	0.075
Group	−1.970	53.5	−0.624	0.535	4.329	50.7	1.138	0.261
Time*Group (Linear)	−1.374	123.9	−0.461	0.645	0.468	119.4	0.155	0.877
Time*Group (Quadratic)	2.700	122.8	0.893	0.374	1.964	118.4	0.640	0.523

^a^Depression Anxiety Stress Scales (DASS-21); ^b^IBD symptoms scale (IBDSS); ^c^Self-Compassion Scale (SCS); ^d^Comprehensive Assessment of Acceptance and Commitment Therapy Processes (CompACT); ^e^Chronic illness-related shame Scale (CISS); ^f^Work and Social Adjustment Scale (WSAS); ^g^Inflammatory Bowel Disease Questionnaire (IBDQ-UK); ^h^EUROHIS-QOL-8.

* *p* < 0.05, ** *p* < 0.01.

#### 3.2.3 Simple effects analyses

From T0 to T1, the EG experienced a reduction in symptoms of anxiety and stress, but this effect did not persist beyond this period (Table 4).

#### 3.2.4 Exploratory factorial ANOVA – DASS-21 severity classification

We observed no between-groups differences in symptoms at T1 after adjusting for T0 symptom scores for depression [*F*_(1,13)_ = 3.613, *p* = 0.08, *η_p_^2^* = 0.217] or for stress [*F*_(1,4)_ = 1.65, *p* = 0.268, *η_p_^2^* = 0.292]. However, a significant difference in T1 between-groups anxiety symptoms [*F*_(1,13)_ = 7.56, *p* = 0.017, *η_p_^2^* = 0.368] was found.

#### 3.2.5 Biomarkers

Regarding the complete blood count, including the erythrogram, leukogram and platelets, no significant alterations were observed between T0 and T3 in both CG and EG. Minor deviations from the reference range, without clinical relevance, were observed for a few patients and are described in detail in the Supplementary Figure S2. Also, no significant differences were observed between T0 and T3 for gender-matched groups from both groups. In the CG, the mean value of albumin significantly increased between T0 and T3 (*p* = 0.004), whereas in the EG, there was a trend suggesting an increase of serum albumin 1 year after the intervention (T3) but without statistical significance (*p* = 0.066) (Supplementary Figure S3). No significant differences were observed in serum CRP nor faecal calprotectin between T0 and T3 in either CG or EG (Supplementary Figures S4, S5).

Results from reliable change indices (RCIs) analyses, did not provide evidence of any particular subgroup of participants who managed to obtain clinically significant improvements over time.

## 4 Discussion

The present study aimed to investigate the acceptability and preliminary efficacy of a face-to-face, group ACT and compassion-based intervention for people with IBD (LIFEwithIBD) through a two-arm RCT. The results for the intervention acceptability were encouraging, as most participants reported a high level of satisfaction with the intervention and considered it useful. Specifically, all participants stated that participating in the intervention was worthwhile and would recommend it to other people with IBD. These results align with the high acceptability presented by previous studies on ACT and compassion-based interventions in chronic illness populations (Trindade et al., 2020b; Dober et al., 2021; Romano et al., 2023). Patients may particularly well accept these third-wave CBT approaches due to their focus on universal psychological processes rather than on symptoms, which may help decrease stigma around the disease and participation in psychotherapy.

No significant differences in depression, anxiety, and stress were found between the EG and the CG. A possible floor effect due to generally low levels of psychological distress at baseline may explain these results [most participants scored below the DASS-21 cut-offs for moderate depression or anxiety (Lovibond, 1995)]. There may not have been enough room for significant improvement, as would have been the case if participants had started at higher levels of psychological distress. From baseline to post-treatment, the EG experienced a significant reduction in symptoms of anxiety and stress, but this effect did not persist during the follow-up periods. This suggests that including booster sessions following the LIFEwithIBD intervention could potentially help maintain the benefits of the intervention. There is evidence that interventions with booster sessions provide better results at follow-up than those without sessions following the main interventional period (Gearing et al., 2013; Wesner et al., 2015). Alternatively, there might also be room for other types of follow-up material like bibliotherapy or online support. In IBS, for example, Ferreira et al. (2018) used an ACT-based self-help manual to support practice in the long-term with positive gains in outcomes obtained from the intervention being sustained at 6 months follow-up.

Moreover, there were no significant differences between groups concerning secondary outcomes (functional impairment, general and health-related quality of life, chronic illness-related shame, IBD symptoms, self-compassion, and psychological flexibility). These unexpected results suggest that the LIFEwithIBD intervention did not influence these outcomes. This, again, may be linked to the low levels of psychological distress at baseline. A significant decrease in stress symptoms and IBD symptom perception was found over time, but there were no differences between groups in this outcome.

The intervention presented effects on disease activity in participants with Crohn’s Disease but not for participants with ulcerative colitis. Participants with Crohn’s Disease from the EG reported significantly lower Harvey-Bradshaw scores at the first follow-up compared to the CG. Only one study using the Harvey-Bradshaw score as an outcome in a psychological intervention trial has found a similar pattern of reduction in symptoms after a CBT and mindfulness-based intervention (Goren et al., 2022).

To determine if the subset of our participants with higher psychological distress particularly benefited from the LIFEwithIBD intervention, we performed a *post hoc* analysis considering mild to severe DASS-21 scores as contributing factors to the response to the intervention. No between-group differences were observed in depression or stress symptoms at post-treatment after adjusting for baseline symptom scores. A significant difference between groups in anxiety symptoms was observed at post-treatment, with the EG reporting a larger reduction in symptoms than the CG. This result should be interpreted considering two points. First, the LIFEwithIBD intervention does seem effective for reducing psychological distress, but since most participants presented lower levels of distress at baseline, the results did not reach statistical significance. Second, in a *post hoc* analysis, limiting the sample size reduces the statistical power, and only the largest effect sizes are detected. Regarding primary outcomes, depression is near to reaching statistical significance (*p* = 0.08). Further investigation is needed regarding the use of this intervention, particularly in patients who are likely to experience comorbid psychological distress.

In addition to the psychological self-reported evaluation, inflammatory biomarkers were assessed. The serum albumin results were unsurprising since this plasma protein tends to decrease quickly in severely ill patients. All participants in this study were under clinical remission, and, as expected, serum albumin remained within the reference interval in all participants from CG and EG, both at baseline and T3. The same rationale holds true for CRP and faecal calprotectin. Our results showed no significant differences regarding both biomarkers between the CG and EG at T3. Similarly, no differences were observed in the total blood count of participants. These results suggest that, from a bioanalytical point of view, disease activity in our sample remained under control throughout the study. The question remains as to whether the LIFEwithIBD intervention could have an impact on gut microbiota phenotype. Indeed, it would be interesting to characterize the gut microbiota of these patients before and after the intervention to ascertain if the modulation of the microbiota-gut-brain axis could be the mechanism underlying the impact of the treatment on psychological (dis)tress herein observed since similar reports have been made regarding IBS (Halkjær et al., 2018).

Overall, these findings need to be interpreted considering several limitations. Due to this study’s relatively small sample size, further studies are required to test the efficacy of the LIFEwithIBD intervention in larger samples. Similarly to previous RCTs testing group interventions in the IBD context (Mikocka-Walus et al., 2017; Wynne et al., 2019), the study showed a considerable attrition rate. This may be due to the time commitment needed to participate in the intervention, combined with the simultaneous management of different life contexts, the lack of monetary compensation for participating in the study, and the lack of transportation to attend the intervention (most screened patients lived outside Coimbra, in the suburbs or in other cities in Portugal). Furthermore, the stigma associated with mental health still present in Portuguese society, which is regarded as one of the main barriers to access to psychological treatment in Portugal (Palha and Palha, 2016), may have also contributed to the study’s limited enrolment rate. Participants from both groups did not indeed present levels of psychological distress, which might have reduced the sensitivity to changes in the outcome measures. This is suggestive that including patients with higher psychological distress (rather than this being an exclusion criterion, such as in the present study) could be of interest in future studies testing the LIFEwithIBD intervention. Another limitation of this study was the use of self-report questionnaires. Although the efficacy of psychological interventions is often assessed through self-assessment, critics believe that the evaluation and interpretation of treatment efficacy can be impaired by the occurrence of a “response shift,” which could contaminate (reduce or amplify) treatment effects. Also, our findings revealed minor changes in anxiety and stress symptoms that may be interpreted as collateral, since the purpose of ACT, mindfulness and self-compassion-based therapies is to improve quality of life, which in our study was considered a secondary outcome which did not show any change. Furthermore, self-report measures that are not very close to each person’s reality may compromise the results, as these measures may not be sensitive to change. For example, in a study conducted in a sample of patients with fibromyalgia, it was found that the most sensitive instrument to the effects of the intervention was the self-report of longitudinal experiences based on questions about the participants’ daily lives in several weeks before and after of the intervention (de la Coba et al., 2022). Further studies should integrate qualitative methods to complement the assessment of intervention efficacy. Finally, although the EG and CG presented similar demographic characteristics, they presented differences in important outcomes at baseline (EG presented a higher level of self-critical attitude and lower levels of psychological flexibility, which may have influenced results). Furthermore, it is important to consider the circumstances under which the study’s assessments occurred. The post-treatment assessment and the second follow-up coincided with two periods of lockdown in Portugal due to the COVID-19 pandemic. It is difficult to hypothesize their possible effects on this study, as studies show that the effects of lockdown periods on mental health are not the same for each individual (Prati and Mancini, 2021).

Despite these limitations, this is the first study to investigate the preliminary efficacy of psychological and disease outcomes and biomarkers of an integrative face-to-face ACT and compassion-based intervention in the context of IBD. Preliminary findings suggest that the LIFEwithIBD intervention may be better suited for IBD patients presenting with high levels of psychological distress. Given that these patients are more likely to self-exclude from these interventions, future studies should address ways of engaging these patients. One possibility would be the use of non-face-to-face approaches such as online interventions. Further research is needed to reach more comprehensive conclusions.

## Data availability statement

The raw data supporting the conclusions of this article will be made available by the authors, without undue reservation.

## Ethics statement

This study was approved by Ethics Committee of the Coimbra University Hospital (CHUC). It was conducted in accordance with the local legislation and institutional requirements. The participants provided their written informed consent to participate in this study.

## Author contributions

CF: Conceptualization, Data curation, Funding acquisition, Investigation, Methodology, Project administration, Resources, Supervision, Writing – original draft. JP: Data curation, Investigation, Methodology, Resources, Writing – original draft. DS: Conceptualization, Formal analysis, Writing – original draft. SO: Data curation, Investigation, Methodology, Resources, Writing – review & editing. AG: Investigation, Writing – review & editing. NF: Conceptualization, Validation, Writing – review & editing. PL-S: Writing – review & editing. SC: Validation, Writing – review & editing. IM-P: Data curation, Investigation, Writing – review & editing. BR: Data curation, Formal analysis, Investigation, Methodology, Writing – review & editing. FP: Conceptualization, Investigation, Methodology, Resources, Validation, Writing – review & editing. IT: Conceptualization, Funding acquisition, Investigation, Methodology, Project administration, Resources, Supervision, Writing – review & editing.
