# Supplementary material for: Randomized controlled trial of an Acceptance and Commitment Therapy and compassion-based group intervention for persons with inflammatory bowel disease: the LIFEwithIBD intervention
Source: Front Psychol. 2024 May 9;15:1367913. doi: 10.3389/fpsyg.2024.1367913 (PMC11112702; doi:10.3389/fpsyg.2024.1367913)
Supplement: Supplementary file 1 [file Table_1.DOCX]

**Supplementary data**

**Instruments**

Biomarkers^47,48^: complete blood count (quantification of erythrocytes, hemoglobin, hematocrit, mean corpuscular volume (MCV), mean corpuscular hemoglobin concentration (MCHC), mean corpuscular hemoglobin (MCH) and Red Cell Distribution Width (RDW); leukograms (leucocytes, neutrophils, lymphocytes, monocytes, eosinophils and basophils; serum albumin; C Reactive Protein (CRP); fecal calprotectin. Differences of each biomarker were analysed at T0 and T3.

**Data analysis**

*Data integrity.* Normality analysis examining standardised skew and kurtosis statistics revealed no substantial deviations from normality (*O < Zskew and Zkurtosis < 2*), except for DASS Depression and Anxiety, which were mildly positive (Zskew of 3.9 and 4.01, respectively). However, these deviations from normality are considered to be within tolerable levels for ANCOVA^54^, and so no adjustments to either the distribution or the alpha level were performed. From T0 to T1, both treatment conditions experienced attrition, *n = 7* in the EG and *n* = 3 in the CG. Missing data analysis revealed no patterns of missingness nor any predictors of attrition from baseline measures.

*Biomarkers*. The results are expressed as the mean± SEM of the values in each group. Two-way ANOVA followed by Sidak’s multiple comparison test was used to compare fecal calprotectin, CRP, albumin and haematological parameters between T0 and T3. A probability value (p) of less than 0.05 was considered significant. Statistical analysis was performed using GraphPad prism software version 9.0 (San Diego, USA).

**Results**

*Complete blood count*

In the control group, the erythrograms of 14 participants had all parameters within the reference intervals. Three participants exhibited values of either MCV or MCH out of the reference range but with no clinical relevance. One participant had MCV above the upper limit at T0 (104 fl [reference: 80-96 fl]) but not at T3 and MCH was increased both at T0 and T3 (35 pg [reference: 27-33 pg] and 34,9 pg, respectively). Another participant had decreased erythrocytes and increased MCV and MCH both at T0 and T3 (erythrocytes: 3.6x10^12^/L at T0 and T1 [reference: 4.1-5.1x10^12^/L], 104 fl at T0 and 100.3 at T3 and 35 pg at T0 and 35,3 pg at T1). Finally, one participant had a normal hemogram at T0 but at T3 had both MCV and MCH below the lower limit (75 fl and 26,4 pg, respectively). Regarding the experimental group, 14 participants had normal erythrograms, one had non significant alterations on the number of erythrocytes and hematocrit and another patient had MCV and MCH out of the reference range but without clinical significance. One participant had both erythrocyte values and the hematocrit bellow the lower limit at T3 but not at T0 (3.6x10^12^/L and 33,6% [reference: 35-47%]). Another participant had increased MCV (98 fl) and MCH (34 pg) at T0 but not at T3.

No significant differences were observed for hemoglobin between males from control and experimental groups between both time points (*p* = 0,983 and *p* = 0.991, respectively). The same was observed for female participants (*p* = 0,958 and *p* = 0,977, for control and experimental groups, respectively) (Figure 2A).

Regarding the leukograms, in the control group, from 17 participants, 12 had a profile within the reference intervals, while 5 had total leukocytes, neutrophils or eosinophils out of the reference interval but without biological significance. One patient had leukopenia with neutropenia at T1 (3,9 G/L [reference: 4-11 G/L] and 1,98 G/L [reference: 2,1-7,6 G/L]), one patient had a slight neutrophilia at T1 (8,28 G/L), one patient had eosinophilia at T1 (0,42 G/L [reference: <0,20 G/L]), one patient had leukopenia (2,4 G/L at T0 and 2.0 G/L at T1) with neutropenia (0,8 G/L at T0 and 0,66 G/L at T1) at both time points and finally, one participant had leukocytosis with neutrophilica at T0 (13,3 G/L and 11,3 G/L) and neutrophilia at T1 (8,07 G/L). In the experimental group, from 16 participants, 8 had normal leukograms at T0 an T3 while the rest of the patients had total leukocytes or neutrophils insignificantly out of range. One participant had leukopenia (3.8 G/L) with neutropenia (1,56 G/L) at T1 but not at T0. Three participants had eosinophil at T1 but not a T0 (0,57 G/L; 0,46 G/L; 0,37 G/L). One participant had eosinophilia both at T0 (0,9 G/L) and T1 (0,93 G/L); one participant had leukocytosis (12,0 G/L) with neutrophilia (9,27 G/L) at T1 but not at T0. One participant had neutrophilia at T0 (8,3 G/L) but not at T1. Finally, one participant had neutrophilia at T0 (7,7 G/L) and leukocytosis with neutrophilia at T1 (11,5 G/L and 9,15 G/L, respectively) (Figure 2B).

Platelets were within the reference interval in all participants from control group, except for two participants who had thrombocytosis at T0 but not at T3 (500x10^9^/L [reference: 150-400x10^9^/L]) and thrombocytopenia at T3 but not at T0 (64x10^9^/L) (Figure 2C).

To summarize, regarding the complete blood count, no significant differences were observed between T0 and T3 for gender-matched groups from both control and experimental groups.

*Serum albumin*

Serum albumin was considered of interest within the scope if IBD since it may decrease in disease flares. All patients included in the study presented serum albumin levels within the reference interval at T0 and T3. In the control group the mean value of albumin significantly increased between both time points. In the experimental group there is a trend suggesting an increase of serum albumin one year after CBT but without statistical significance The values in the control group ranged from 4,2±0,09 g/dL at T0 and 4,5±0,09 g/dL at T3 (*p* = 0.0038). In this group serum albumin increased in 16 out of 17 participants. In the experimental group there were two participants with unaltered albumin between both time points and one had lower serum levels at T3 in comparison to T0 (**Figure 3**). Nonetheless, the tendency was again to an increase of serum albumin levels from 4,2±0,10 g/dL at T0 to 4,5±0,10 g/dL at T3 (*p* = 0.066).

*C Reactive Protein (CRP)*

No significant differences were observed in serum CRP between T0 and T3 in either the control or experimental group (Figure 4). In the control group, CRP decreased from 5.3±1.13 mg/L at T0 to 4.2±1.15 mg/L at T3 (*p* = 0,878) (Figure 4A). If one considers individual variation, in the control group, CRP remained under the reference limit (< 5 mg/L) between T0 and T3 in 53% of the patients, decreased in 29% and increased in 18% (Figure 4A, inset). Similarly, in the experimental group, serum PCR remained lower than 5 mg/L in 53% of the participants in both T0 and T3, decreased in 27% and increased in 20% (Figure 5B, inset). Also, the mean PCR levels in the experimental group decreased from 6.2±2.38 mg/L at T0 to 5.5±2.09 mg/L at T3 (*p* = 0.952).

*Fecal calprotectin*

No significant differences were observed on fecal calprotectin concentration between T0 and T3 both in the control and experimental groups (Figure 5). In the control group, the mean calprotectin concentration at T0 was 370±93.1μg/g, decreasing to 297±48.7 μg/g at T3 (*p* = 0.165). In the experimental group, there was also a tendency to a decreased fecal calprotectin at T3 (301±96.3 μg/g) in comparison to T0 (397±92.3 μg/g, *p* = 0.678). Again, when analysing in detail both groups, in the controls, four participants had a calprotectin concentration bellow 80 μg/g at both T0 and T1. The participants from the control group who had calprotectin higher than 80 μg/g, 77% had lower concentration at T1 in comparison to T0 (Figure 5A, inset). Regarding the experimental group, three participants had calprotectin values bellow 80 μg/g at both time points. From those who had not, 67% showed a reduction from T0 to T1 (Figure 5B, inset).


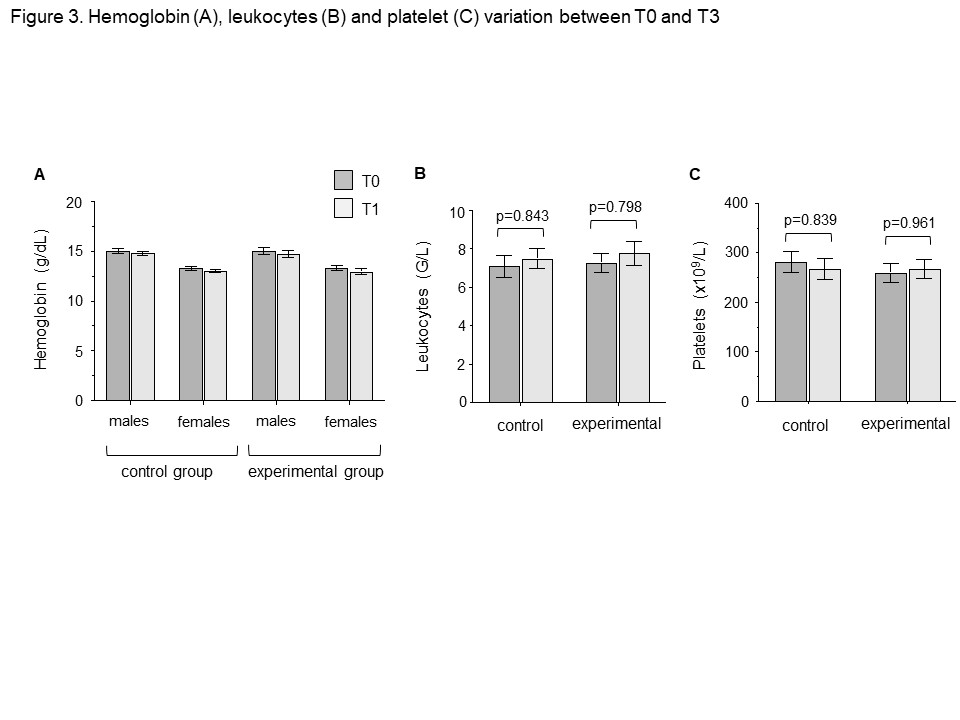
Figure 2. Haemoglobin (A), leukocytes (B) and platelet (C) variation between T0 and T3


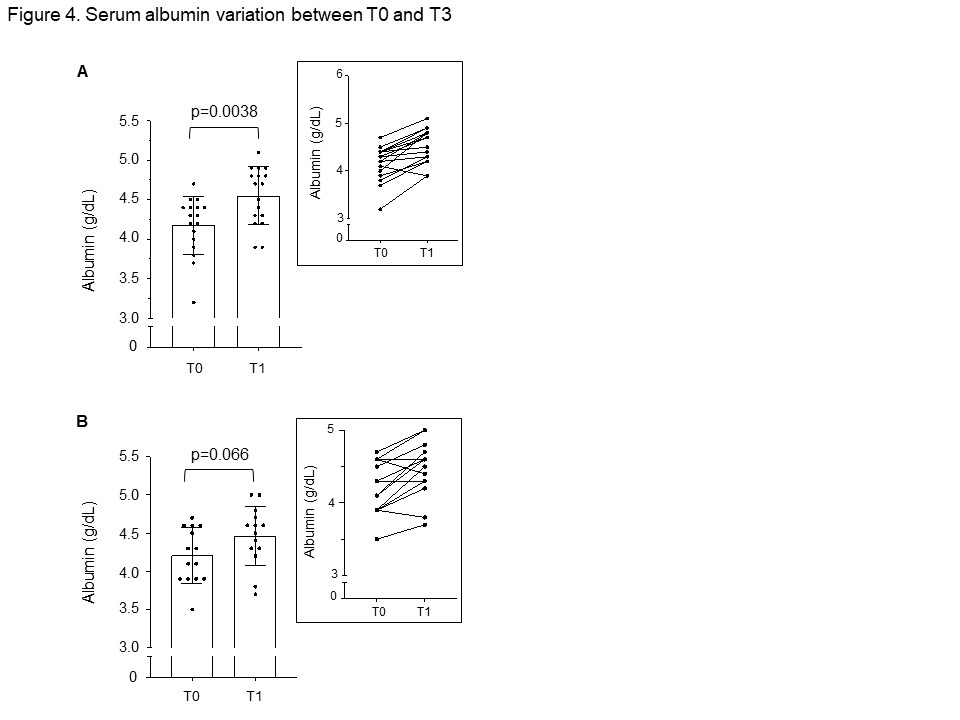
Figure 3. Serum albumin variation between T0 and T3


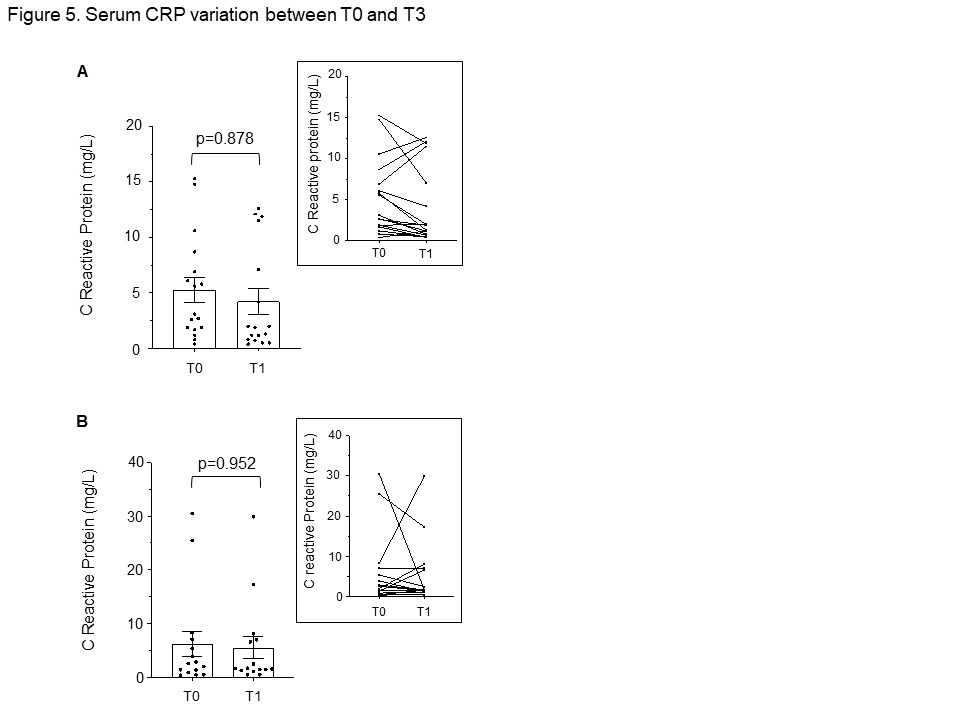
Figure 4. Serum CRP variation between T0 and T3


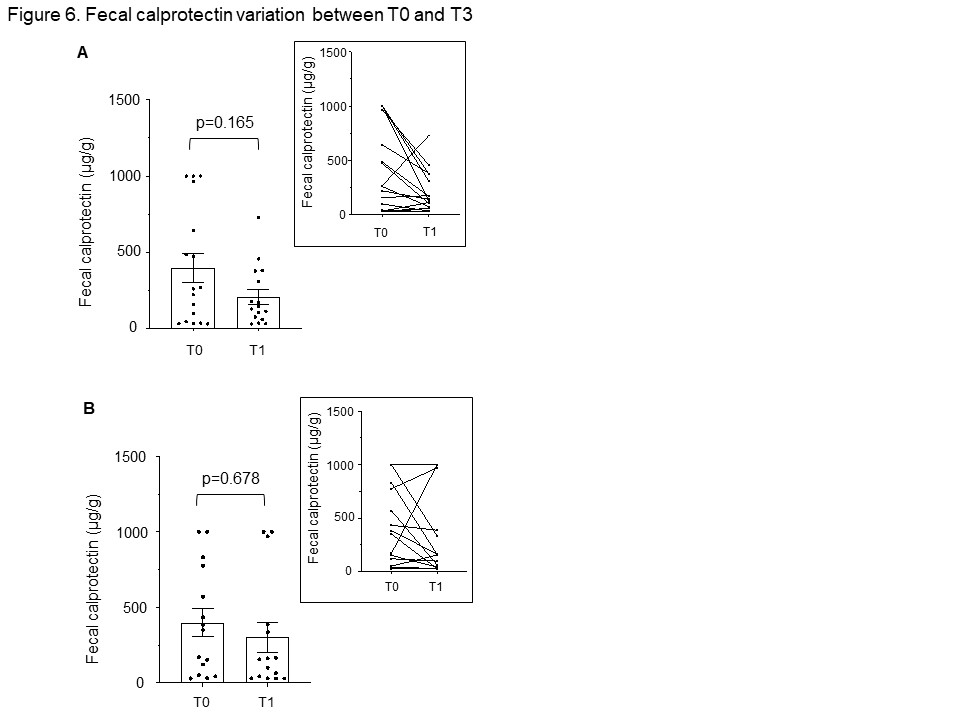
Figure 5. Fecal calprotectin variation between T0 and T3
